# Factors Associated with Antenatal Influenza Vaccination in a Medically Underserved Population

**DOI:** 10.1155/2020/5803926

**Published:** 2020-01-27

**Authors:** Jenna C. Adams, Hope H. Biswas, Sheree L. Boulet, Kamini Doraivelu, Michele K. Saums, Lisa Haddad, Denise J. Jamieson

**Affiliations:** ^1^Emory University School of Medicine, Atlanta, GA, USA; ^2^Department of Gynecology and Obstetrics, Emory University School of Medicine, Atlanta, GA, USA; ^3^Rollins School of Public Health, Emory University, Atlanta, GA, USA

## Abstract

Influenza infection in pregnant women is associated with increased risk of morbidity and mortality. Despite recommendations for all women to receive the seasonal influenza vaccine during pregnancy, vaccination rates among pregnant women in the U.S. have remained around 50%. The objective of this study was to evaluate clinical and demographic factors associated with antenatal influenza vaccination in a medically underserved population of women. We conducted a retrospective cohort study at Grady Memorial Hospital, a large safety-net hospital in Atlanta, Georgia, from July 1, 2016, to June 30, 2018. Demographic and clinical characteristics were abstracted from the electronic medical record. The Kotelchuck index was used to assess prenatal care adequacy. Relative risks and 95% confidence intervals for associations between receipt of influenza vaccine and prenatal care adequacy, demographic characteristics, and clinical characteristics were calculated using multivariable log-binominal models. Among 3723 pregnant women with deliveries, women were primarily non-Hispanic black (68.4%) and had Medicaid as their primary insurance type (87.9%). The overall vaccination rate was 49.8% (1853/3723). Inadequate prenatal care adequacy was associated with a lower antenatal influenza vaccination rate (43.5%), while intermediate and higher levels of prenatal care adequacy were associated with higher vaccination rates (66.9–68.3%). Hispanic ethnicity, non-Hispanic other race/ethnicity, interpreter use for a language other than Spanish, and preexisting diabetes mellitus were associated with higher vaccination coverage in multivariable analyses. Among medically underserved pregnant women, inadequate prenatal care utilization was associated with a lower rate of antenatal influenza vaccination. Socially disadvantaged women may face individual and structural barriers when accessing prenatal care, suggesting that evidenced-based, tailored approaches may be needed to improve prenatal care utilization and antenatal influenza vaccination rates.

## 1. Introduction

Pregnant women are at increased risk of severe influenza infection compared to the general population [1]. In the most recent 2009 H1N1 influenza pandemic, pregnant women represented a disproportionate number of influenza-related deaths compared to the general population [1–3]. There is also evidence of increased rates of hospital admissions during influenza seasons among pregnant women compared to nonpregnant women [4, 5]. Current recommendations from the Centers for Disease Control and Prevention (CDC) Advisory Committee on Vaccination Practices (ACIP) [6] and the American College of Obstetricians and Gynecologists (ACOG) [7] support routine influenza vaccination of pregnant women regardless of trimester. Despite these recommendations, seasonal influenza vaccine uptake among pregnant women continues to be well below the Healthy People 2020 objective of 80% [8, 9]. Nationally, there are disparities in seasonal influenza vaccination in pregnant women based on a patient's age, race and ethnicity, level of education, marital status, insurance coverage, employment status, income level, and comorbid health conditions [9].

Safety-net hospitals provide care for medically underserved patients who are likely to have multiple compounding social determinants of health that affect their access to and receipt of recommended health care services, including influenza vaccination [10]. While receiving care at a safety-net hospital does not necessarily categorize an individual as medically underserved, we assume that the majority of patients who receive care at safety-net hospitals do so because they have limited or no access to care at other health institutions [11]. This population represents an ideal group in which to study the effect of adequacy of prenatal care on influenza vaccination status because these women are more likely to experience barriers to care.

The primary objective of this study was to determine whether adequacy of prenatal care is associated with antenatal influenza vaccination in a population of medically underserved pregnant women. We hypothesized that less than adequate prenatal care is associated with decreased likelihood of antenatal influenza vaccination compared to adequate prenatal care (defined as attending 80% or more of recommended prenatal care visits). Our secondary objective was to determine if selected demographic and clinical characteristics are associated with antenatal influenza vaccination in this population.

## 2. Materials and Methods

We conducted a retrospective cohort study of pregnant women who delivered at Grady Memorial Hospital under the supervision of Emory University clinicians. Grady Memorial Hospital, which is part of Grady Health System, is the only public safety-net hospital in Atlanta, Georgia [10]. Emory University clinicians provide approximately 80% of patient care at Grady Memorial Hospital. Only deliveries to Emory University clinicians were included in the study due to challenges with obtaining delivery data from outside institutions. We included all women who delivered one or more fetuses after twenty weeks of gestation, regardless of the viability of the fetus, from July 1, 2016, to June 30, 2018, to include two full influenza seasons (2016–17 and 2017–18). We will describe this population henceforth as “women with deliveries”.

To identify women with deliveries, we used two data sources: hospital charge data from the Emory Medical Care Foundation database, which records all billing transactions at Grady Memorial Hospital by Emory University clinicians, and a delivery record in the labor and delivery suite at Grady Memorial Hospital. For both data sources, deliveries were confirmed by reviewing obstetrical documentation in the electronic health record (EHR).

The EHRs of all patients identified were manually reviewed for demographic information. Clinical characteristics, including parity, substance use in pregnancy, history of chronic diseases, prenatal care utilization, and influenza vaccination status, were also abstracted. Study data were collected and managed using Research Electronic Data Capture (REDCap) tools hosted at Emory University [12]. All researchers were trained in EHR abstraction by the developer of the REDCap instrument (JCA) and used a codebook to ensure consistent methodology.

Prenatal care adequacy was defined using the Kotelchuck index [13]. By calculating the ratio of number of visits attended to ACOG's recommended number of visits, patients were grouped into four categories: inadequate (less than 50%, including no prenatal care), intermediate (50–79%), adequate (80–109%), and adequate plus (greater than or equal to 110%) [13]. Prenatal care was defined as “unknown” if patients received some or all prenatal care outside of Grady Health System, which prohibited quantification of adequacy of care with the Kotelchuck index.

Influenza vaccination was defined as receipt of influenza vaccine of any type that occurred from twelve months before delivery to the delivery date. Receipt of influenza vaccine was captured in the EHR in three ways: vaccines given within Grady Health System, vaccines received in the state of Georgia reported to the Georgia Registry of Immunization Transactions and Services, and vaccines self-reported by patients to their prenatal care provider documented in a prenatal care note. The influenza vaccination rate among pregnant women was calculated by dividing the number of women with documented influenza vaccination in our population divided by the number of women with a delivery in each year-long period.

We used *χ*^2^ tests to compare distributions of selected demographic and clinical characteristics for women who did and did not receive the influenza vaccine. These characteristics included age, race/ethnicity, interpreter use, parity, chronic medical conditions, tobacco use in pregnancy, prenatal care adequacy, and primary insurance type. Chronic medical conditions including asthma, cardiovascular disease excluding essential hypertension, diabetes mellitus, and HIV-positive status were included due to their association with increased morbidity with influenza infection [6]. We used multivariable log-binomial regression models to estimate unadjusted and adjusted relative risks (aRR) and 95% confidence intervals (CI) for associations between receipt of influenza vaccine and prenatal care adequacy, demographic characteristics, and clinical characteristics. Generalized estimating equations were used to account for clustering among women with multiple deliveries during the study period. To evaluate the effect of including patients with unknown prenatal care utilization, we conducted a sensitivity analysis restricting the study population to patients with known prenatal care utilization. *P* < 0.05 was considered statistically significant. Statistical analyses were conducted using Statistical Analysis Software (SAS Institute Inc., Cary, NC), version 9.4.

This study was approved by the institutional review board at Emory University and the Grady Research Oversight Committee.

## 3. Results

From July 1, 2016, to June 30, 2018, we identified 3723 women with deliveries, in which the overall influenza vaccination rate was 49.8%. The influenza vaccination rate was stable across the 2016–17 and 2017–18 seasons (49.1% July 1, 2016–June 30, 2017, 50.5% July 1, 2017–June 30, 2018).

As shown in Table 1, our study population was primarily non-Hispanic black (68.4%). Approximately 20% of the women in our population were Hispanic with Spanish being the most common language utilized for interpretive services. The majority of women were multiparous with nearly half of women reporting a parity of three or more. Very few women had preexisting cardiovascular disease, diabetes mellitus, or HIV. Eleven percent of women reported using tobacco products during pregnancy. Medicaid was the payer for the majority of deliveries.

Women with intermediate, adequate, or adequate plus prenatal care utilization had the highest vaccine coverage levels (66.9-68.3%, Table 2). Vaccination rates were also high among Hispanic women, non-Hispanic other race women, women who used an interpreter for Spanish, and women with preexisting diabetes mellitus (63.9–64.6%). Vaccination rates were the lowest among non-Hispanic white women, women who used tobacco products during pregnancy, women without medical insurance, women with Medicare insurance, and women with unknown prenatal care adequacy (22.1–38.9%).

In the adjusted model, Hispanic ethnicity, non-Hispanic other race/ethnicity, and interpreter use for a language other than Spanish were associated with an increased likelihood of antenatal influenza vaccination. Preexisting diabetes mellitus was also associated with increased antenatal influenza vaccination compared to the reference group, while tobacco use in pregnancy was associated with a decreased antenatal influenza vaccination compared to the reference group. Inadequate prenatal care was associated with a 30% decreased likelihood of influenza vaccination (aRR 0.68, 95% CI 0.63–0.73), and unknown prenatal care adequacy was associated with a nearly 70% decreased likelihood of influenza vaccination compared to adequate prenatal care (aRR 0.33, 95% CI 0.28–0.40).

Excluding women with unknown prenatal care, 51.9% received inadequate prenatal care, 17.8% received intermediate prenatal care, 22.4% received adequate prenatal care, and 8.0% received adequate plus prenatal (data not shown). Adjusted relative risks in this restricted population were similar to the full study population (supplemental table (available here)).

## 4. Discussion

The overall vaccination rate in our study (48.9%) is similar to that reported by the CDC in national data for the 2016-17 and 2017-18 seasons, which was approximately 50% [9, 14]. Our finding of inadequate prenatal care being associated with decreased likelihood of influenza vaccination is consistent with results from state- and national-level Pregnancy Risk Assessment Monitoring System studies [15, 16]. Our findings of similar vaccination rates for intermediate, adequate, and adequate plus prenatal care suggest a threshold effect of increasing prenatal care in relation to influenza vaccine uptake in our study population. This is a novel finding compared to similar studies which have noted a dose-dependent relationship between prenatal care adequacy and influenza vaccine coverage [15, 16]. Given the high percentage of women in our population that received less than adequate prenatal care, we speculate that providers in this setting may recognize the limited opportunities for vaccination and prioritize influenza vaccine counseling during all prenatal care visits.

We were unable to quantify 16.2% of our study population's prenatal care adequacy because these women reported received prenatal care from providers outside of Grady Health System. A sensitivity analysis testing the effect of excluding this unknown group demonstrated comparable relative risks, which eliminated concern about the inclusion of these women biasing our associations between prenatal care adequacy and influenza vaccination. We found that this unknown group had significantly decreased vaccination rates compared to those with adequate prenatal care. While some women may have received vaccines in another state or country, transferring care during pregnancy may inherently be associated with decreased risk of antenatal influenza vaccination and may reflect missed opportunities for vaccine counseling.

Our findings of increased vaccination rates among Hispanic women and non-Hispanic other race women are consistent with those from a recent nationally representative study on race/ethnicity and influenza vaccination during pregnancy [16]. We also identified that women who used interpreters for languages other than Spanish had increased rates of influenza vaccination compared to women who used no interpreter, which is a novel finding and may warrant future study, particularly regarding the impact of interpreters' own vaccination beliefs on the likelihood of vaccine uptake among patients. Together, our data further support low vaccination rates in non-Hispanic black women compared with high vaccination rates in Hispanic and non-Hispanic other race women. We speculate that community-level factors, such as the circulation of myths surrounding the influenza vaccine, social norms, and the level of trust in medical recommendations, may influence the differential uptake of antenatal influenza vaccine in these racial and ethnic groups [17, 18].

We found that women with preexisting diabetes mellitus were more likely to receive recommended antenatal influenza vaccination. These results are supported by CDC surveillance data showing higher vaccination rates among women with high-risk conditions and among adults with chronic medical conditions in the general population [9, 19]. Diabetes mellitus increases an individual's risk of severe complications from influenza and also necessitates frequent visits with a maternal-fetal medicine physician during pregnancy [6], factors that may affect both the quantity and quality of counseling that these patients receive concerning influenza vaccination. HIV-positive women, who are similar to women with preexisting diabetes mellitus with respect to their increased risk of influenza complications and recommended frequent visits with a maternal-fetal medicine physician, did not have a statistically significant increased rate of influenza vaccination. This could be due to competing priorities in the time-limited prenatal care appointment or underlying differences in the attitudes of these patient populations toward influenza vaccine. Tobacco use was associated with a decreased likelihood of receiving an influenza vaccine during pregnancy, which has been previously reported [16, 20, 21].

The strengths of the current study include examining a large, medically underserved population accessing care at a safety-net hospital over a period of two influenza seasons. We were able to account for influenza vaccination in a number of ways. The limitations of our study include being unable to examine other social determinants of health, such as education, socioeconomic status, marital status, employment, nativity, and citizenship status, which were not systematically recorded in the EHR and may be important predictors of maternal vaccination. We were also limited by only assessing pregnant women with a delivery rather than all pregnant women (regardless of pregnancy outcome) and were only able to include those who were delivered by Emory providers rather than all deliveries at Grady Memorial Hospital. Our study may be limited in its generalizability to other safety-net hospitals or similar institutions that care for medically underserved patients. Our inability to abstract data on out-of-state vaccinations and the possible underreporting of influenza vaccines to the Georgia Registry of Immunization Transactions and Services may have underestimated our vaccination rates [22], while inclusion of self-report as a measure of vaccination status may have overestimated our vaccination rates [23].

## 5. Conclusions

Our study highlights prenatal care adequacy as a factor that could be targeted in an effort to increase influenza vaccination rates among medically underserved pregnant women. Providers should prioritize recommending, counseling, and offering influenza vaccine at each prenatal care appointment to reduce the impact of barriers to vaccination among women who have few prenatal visits. Evidence-based system interventions, including standing orders, home visits, and patient reward incentives, may specifically benefit organizations caring for women who are medically underserved. Improving the accuracy and scope of vaccination reporting may also permit providers to easily identify patient's vaccination status and facilitate systematic vaccine recommendation and offering.

## Figures and Tables

**Table 1:** Demographic and clinical characteristics of pregnant women with deliveries by influenza vaccination status, July 1, 2016–June 30, 2018.

Characteristic	Total study population *n* = 3723	No influenza vaccination *n* = 1870 (50.2%)	Influenza vaccination *n* = 1853 (49.8%)
Age at delivery (years)			
<21	571 (15.3)	308 (16.5)	263 (14.2)
21–34	2594 (69.7)	1296 (69.3)	1298 (70.1)
>34	558 (15.0)	266 (14.2)	292 (15.8)
Race/ethnicity			
Black, non-Hispanic	2529 (68.4)	1405 (75.7)	1124 (61.1)
Hispanic	834 (22.6)	299 (16.1)	535 (29.1)
Other, non-Hispanic	216 (5.9)	78 (4.2)	138 (7.5)
White, non-Hispanic	116 (3.1)	73 (3.9)	43 (2.3)
Interpreter use			
No interpreter	2872 (77.1)	1552 (83.0)	1320 (71.2)
Spanish	570 (15.3)	202 (10.8)	368 (19.9)
Other language	281 (7.6)	116 (6.2)	165 (8.9)
Parity			
0	868 (23.3)	444 (23.8)	424 (22.9)
1	279 (7.5)	132 (7.1)	147 (7.9)
2	901 (24.2)	424 (22.7)	477 (25.7)
≥3	1673 (45.0)	868 (46.5)	805 (43.4)
Chronic medical condition			
Asthma	357 (9.6)	185 (9.9)	172 (9.3)
Cardiovascular disease	33 (0.9)	18 (1.0)	15 (0.8)
Diabetes mellitus	96 (2.6)	34 (1.8)	62 (3.3)
HIV-positive status	81 (2.2)	34 (1.8)	47 (2.5)
Tobacco use in pregnancy	414 (11.4)	260 (14.2)	154 (8.6)
Prenatal care adequacy			
Inadequate	1614 (43.5)	912 (49.0)	702 (38.0)
Intermediate	553 (14.9)	183 (9.8)	370 (20.0)
Adequate	696 (18.7)	221 (11.9)	475 (25.7)
Adequate plus	249 (6.7)	79 (4.2)	170 (9.2)
Unknown	601 (16.2)	468 (25.1)	133 (7.2)
Primary insurance type			
Self-pay	229 (6.2)	40 (7.5)	89 (4.8)
Medicaid	3275 (87.9)	1613 (86.3)	1662 (89.6)
Medicare	19 (0.5)	13 (0.7)	6 (0.3)
Commercial	201 (5.4)	104 (5.6)	97 (5.2)

Influenza vaccination was defined as documented administration of influenza vaccine in the Grady Health System EHR, the Georgia Registry of Immunization Transactions and Services, or self-reported receipt of influenza vaccine within the time frame of twelve months before delivery and the delivery date. Other, non-Hispanic race/ethnicity includes Asian, Hawaiian/other Pacific Islander, Native American, and multiple races. Data are *n* (%). Data were missing for <5% of all variables.

**Table 2:** Factors associated with antenatal influenza vaccination among pregnant women with deliveries.

Characteristic	Vaccination rate (%)	Vaccination unadjusted RR (95% CI)	Vaccination adjusted RR (95% CI)
Age at delivery (years)			
<21	46.1	0.92 (0.84–1.01)	0.98 (0.88–1.09)
21–34	50.0	ref	ref
>34	52.3	1.05 (0.96–1.14)	1.00 (0.91–1.09)
Race/ethnicity			
Black, non-Hispanic	44.4	ref	ref
Hispanic	64.2	1.44 (1.35–1.54)^∗^	1.38 (1.24–1.52)^∗^
Other, non-Hispanic	63.9	1.44 (1.29–1.60)^∗^	1.21 (1.07–1.38)^∗^
White, non-Hispanic	37.1	0.83 (0.66–1.06)	1.06 (0.84–1.33)
Interpreter use			
No interpreter	46.0	ref	ref
Spanish	64.6	1.40 (1.31–1.51)^∗^	1.00 (0.90–1.12)
Other language	58.7	1.28 (1.15–1.42)^∗^	1.17 (1.04–1.32)^∗^
Parity			
0	48.9	ref	ref
1	52.7	1.08 (0.95–1.23)	1.06 (0.94–1.21)
2	52.9	1.08 (0.99–1.19)	1.05 (0.96–1.16)
≥3	48.1	0.99 (0.91–1.07)	0.96 (0.87–1.06)
Chronic medical condition			
Asthma	48.2	0.96 (0.86–1.08)	1.06 (0.94–1.19)
Cardiovascular disease	45.5	0.91 (0.63–1.33)	1.12 (0.78–1.59)
Diabetes mellitus	64.6	1.31 (1.12–1.52)^∗^	1.29 (1.11–1.51)^∗^
HIV-positive status	58.0	1.17 (0.97–1.41)	1.18 (0.97–1.42)
Tobacco use in pregnancy	37.2	0.73 (0.64–0.83)^∗^	0.85 (0.75–0.97)^∗^
Prenatal care adequacy			
Inadequate	43.5	0.64 (0.59–0.69)^∗^	0.68 (0.63–0.73)^∗^
Intermediate	66.9	0.98 (0.91–1.06)	1.00 (0.92–1.09)
Adequate	68.3	ref	ref
Adequate plus	68.3	1.00 (0.91–1.10)	1.00 (0.90–1.11)
Unknown	22.1	0.32 (0.28–0.38)^∗^	0.33 (0.28–0.40)^∗^
Primary insurance type			
Self-pay	38.9	0.81 (0.65–1.00)	1.07 (0.86–1.32)
Medicaid	50.7	1.05 (0.91–1.22)	1.09 (0.93–1.28)
Medicare	31.6	0.65 (0.33–1.29)	0.76 (0.38–1.51)
Private	48.3	ref	ref

Influenza vaccination was defined as documented administration of influenza vaccine in the Grady Health System EHR, the Georgia Registry of Immunization Transactions and Services, or self-reported receipt of influenza vaccine within the time frame of twelve months before delivery and the delivery date. Data are %, relative risk (95% confidence interval), and adjusted relative risk (95% confidence interval); ^∗^*P* < 0.05. Other, non-Hispanic race/ethnicity includes Asian, Hawaiian/other Pacific Islander, Native American, and multiple races.

## Data Availability

The medical record data used to support the findings of this study have not been made available because they contain protected health information that could be used to identify individuals.

## Supplementary Materials

Supplementary Materials Supplemental Table: factors associated with antenatal influenza vaccination among pregnant women with deliveries, excluding women with unknown prenatal care.
