# Supplementary material for: Factors Associated with Antenatal Influenza Vaccination in a Medically Underserved Population
Source: Infect Dis Obstet Gynecol. 2020 Jan 27;2020:5803926. doi: 10.1155/2020/5803926 (PMC7204164; doi:10.1155/2020/5803926)
Supplement: Supplementary Materials — Supplemental Table: factors associated with antenatal influenza vaccination among pregnant women with deliveries, excluding women with unknown prenatal care. [file 5803926.f1.pdf]

Supplemental Table: Predictors of Antenatal Influenza Vaccination Among Pregnant Women with Deliveries, Excluding Women with Unknown Prenatal Care

Characteristic	Vaccination Adjusted RR (95% CI)
Age at delivery (years)	
<21	0.94 (0.85–1.05)
21–34	ref
>34	0.99 (0.90–1.08)
Race/ethnicity	
Black, non-Hispanic	ref
Hispanic	1.35 (1.22–1.49)*
Other, non-Hispanic	1.15 (1.01–1.30)*
White, non-Hispanic	0.97 (0.76–1.21)
Interpreter use	
No interpreter	ref
Spanish	1.02 (0.91–1.13)
Other language	1.22 (1.09–1.38)*
Parity	
0	ref
1	1.09 (0.96–1.24)
2	1.06 (0.96–1.17)
≥3	0.97 (0.88–1.07)
Chronic medical condition	
Asthma	1.03 (0.92–1.16)
Cardiovascular disease	1.21 (0.86–1.72)
Diabetes mellitus	1.24 (1.07–1.45)*
HIV-positive status	1.17 (0.96–1.41)
Tobacco use in pregnancy	0.86 (0.76–0.98)*
Prenatal care adequacy	
Inadequate	0.67 (0.62–0.73)*
Intermediate	1.00 (0.92–1.08)
Adequate	ref
Adequate plus	1.00 (0.90–1.11)
Primary insurance type	
Self-pay	1.17 (0.95–1.45)
Medicaid	1.09 (0.93–1.29)
Medicare	0.82 (0.41–1.62)
Private	ref

Influenza vaccination was defined as documented administration of influenza vaccine in the Grady Health System EHR, the Georgia Registry of Immunization Transactions and Services, or self-reported receipt of influenza vaccine within the time frame of twelve months before delivery and the delivery date.

Data are %, relative risk (95% confidence interval), and adjusted relative risk (95% confidence interval. * P <0.05

Other, non-Hispanic race/ethnicity includes Asian, Hawaiian/other Pacific Islander, Native American, and multiple races.
